# Recruitment of Nursing Home Residents for Randomized Control Study: Challenges and Strategies

**DOI:** 10.1093/geroni/igaf122.3036

**Published:** 2025-12-31

**Authors:** Diana Sturdevant, Julie Gordon, Barbara Carlson

**Affiliations:** The University of Oklahoma Health Sciences Center, Oklahoma City, Oklahoma, United States; The University of Oklahoma Fran and Earl Ziegler College of Nursing, Oklahoma City, Oklahoma, United States; The University of North Carolina at Wilmington, Wilmington, North Carolina, United States

## Abstract

Numerous studies attest to the challenges of recruiting and retaining nursing home (NH) residents in clinical trials. In this presentation, we present methods we used to recruit and retain NH residents in a randomized, double-blinded, comparative study that assessed the effects of the Shingrix vaccine on the innate immune system residents (age 65-XXX) during the COVID-19 pandemic. The protocol was quite complex (1) recruitment/consent, (2) Baseline blood draw and 1st injection, and (3) a second injection/blood draw (3 months later). The post-treatment control group had the same number of visits but had two additional visits to receive the vaccine. Of the 217 residents enrolled in the study, 160 residents from 23 NHs completed the study. Our success relied upon working alongside corporate and facility leaders to tailor our protocols to the ever-evolving clinical landscape, which included (1) fluctuating trends in staff/resident outbreaks, (2) weather emergencies, (3) the introduction of new local/federal government regulations (i.e., regular COVID testing, rules governing entry into a facility), and (4) introduction of the COVID vaccine/vaccine hesitancy. While corporate endorsement helped the team to gain entrance, our strategies enabled nursing home staff to become vested in the research. Recognizing the value, both socially and medically, the protocols had for their clients, they worked with us to recruit and retain resident research participants.

